# Estimating the costs of genomic sequencing in cancer control

**DOI:** 10.1186/s12913-020-05318-y

**Published:** 2020-06-03

**Authors:** Louisa G. Gordon, Nicole M. White, Thomas M. Elliott, Katia Nones, Anthony G. Beckhouse, Astrid J. Rodriguez-Acevedo, Penelope M. Webb, Xing J. Lee, Nicholas Graves, Deborah J. Schofield

**Affiliations:** 1 QIMR Berghofer Medical Research Institute, Population Health Department, 300 Herston Rd, Brisbane, QLD 4006 Australia; 2 Queensland University of Technology (QUT), School of Nursing, Institute of Health and Biomedical Innovation, Brisbane, Queensland Australia; 3 The University of Queensland, School of Public Health, Brisbane, Queensland Australia; 4 Queensland University of Technology (QUT), Australian Centre for Health Services Innovation, Institute of Health and Biomedical Innovation, Brisbane, Queensland Australia; 5 QIMR Berghofer Medical Research Institute, Medical Genomics Laboratory, Brisbane, Queensland Australia; 6 Macquarie University, Centre for Economic Impacts of Genomic Medicine, Department of Economics, Sydney, Australia; 7 BGI Australia, 300 Herston Rd, Herston, Brisbane, Q4006 Australia

**Keywords:** Genomic sequencing, Cancer, Cost-analysis, Micro-costing

## Abstract

**Background:**

Despite the rapid uptake of genomic technologies within cancer care, few studies provide detailed information on the costs of sequencing across different applications. The objective of the study was to examine and categorise the complete costs involved in genomic sequencing for a range of applications within cancer settings.

**Methods:**

We performed a cost-analysis using gross and micro-costing approaches for genomic sequencing performed during 2017/2018 across different settings in Brisbane, Australia. Sequencing was undertaken for patients with lung, breast, oesophageal cancers, melanoma or mesothelioma. Aggregated resource data were captured for a total of 1433 patients and point estimates of per patient costs were generated. Deterministic sensitivity analyses addressed the uncertainty in the estimates. Estimated costs to the public health system for resources were categorised into seven distinct activities in the sequencing process: sampling, extraction, library preparation, sequencing, analysis, data storage and clinical reporting. Costs were also aggregated according to labour, consumables, testing, equipment and ‘other’ categories.

**Results:**

The per person costs were AU$347–429 (2018 US$240–297) for targeted panels, AU$871–$2788 (2018 US$604–1932) for exome sequencing, and AU$2895–4830 (2018 US$2006-3347) for whole genome sequencing. Cost proportions were highest for library preparation/sequencing materials (average 76.8% of total costs), sample extraction (8.1%), data analysis (9.2%) and data storage (2.6%). Capital costs for the sequencers were an additional AU$34–197 (2018 US$24–67) per person.

**Conclusions:**

Total costs were most sensitive to consumables and sequencing activities driven by commercial prices. Per person sequencing costs for cancer are high when tumour/blood pairs require testing. Using the natural steps involved in sequencing and categorising resources accordingly, future evaluations of costs or cost-effectiveness of clinical genomics across cancer projects could be more standardised and facilitate easier comparison of cost drivers.

## Background

Sequencing the human genome using the first-generation Sanger sequencing techniques from 1977 took nearly 15 years, required extensive collaboration between hundreds of laboratories around the world and was reported to cost US$100 million [1]. Next-generation sequencing from 2005 overcame much of the earlier problems by producing massively parallel sequencing of multiple samples and high throughput at a fraction of the cost (US$1 million) and time (2 months) [1]. These technologies and sequencing platforms continue to evolve. Genomic sequencing is increasingly used in clinical medicine to provide important information to guide patient care [2, 3]. Genomic sequencing goes beyond genetic testing of single genes by sequencing either large numbers of genes (targeted panels), all protein-coding regions of the genome (whole exome sequencing) or the complete genome (whole genome sequencing). Like all new medical technologies, genomic sequencing technologies need to be evaluated for their health, social and economic impacts before being widely implemented [4, 5].

There is significant interest in genomic sequencing in oncology due to its potential to advance diagnostics and personalise treatments, as well as to improve our understanding of treatment responses and the causes and mechanisms of tumour development. Clinical genetic testing for gene panels is routinely occurring in melanoma of the skin, colorectal cancers, and breast cancers [6]. The natural extension is to undertake genomic sequencing to provide a more comprehensive assessment of pathogenic variants [7] from which to make decisions to change patient care.

Genomic sequencing for medical diagnosis and decision-making is often considered for public funding within an economic evaluation framework [8–11]. Economic evaluations assess the costs and patient outcomes of sequencing applications and compare these with usual clinical practice without sequencing. In a full economic evaluation, the costs of genomic sequencing, the subsequent changes to patient management and all downstream consequences would be examined [12]. Assessing the value of genomic sequencing is challenging due to the inherent complexity of the sequencing process itself and the need to assess the impact of actioning subsequent findings on patient management.

Examining the costs of genomic sequencing in patients with cancer is important because the process is more complex in cancer than in other diseases. Cancer genomics often involves sequencing both tumour and germline (blood, saliva etc) samples to identify pathogenic variants. A recent Canadian study analysed patient-level data over 3 years on a mixed cancer cohort and found whole genome sequencing costs of CA$34,886 (2018 US$27,227) per patient [13] while costs were CA$5519 (2018 US$4307) [14] per patient for autism spectrum disorder and CA$2851 (2018 US$2225) [15] for paediatric conditions. A recent micro-costing study by Schwarze et al. (2020) shows whole genome sequencing was £6841 per cancer case and £7050 per rare disease case (approx. US$5700), showing similar costs across cancer and other diseases [16]. Exome sequencing costs in various cancer and non-cancer patient groups are in the range of US$1292 to $3594 per patient [14, 17, 18]. These compare with per-patient costs of targeted panels of known clinical variants for patients with cancer of US$695 to $2861 (2018 prices) [19–22].

With the improvements in genomic sequencing technologies, automation processes and computing, the price of genomic sequencing is falling and some claim it has reached the ‘$1000 genome’ level [23]. Recent micro-costing studies of genomic sequencing are reported in the United Kingdom [16], France [22], Canada [13, 14, 19], The Netherlands [23], US [17] and Germany [24] and provide useful information on sequencing resources. These studies present costs for different units of analysis (per patient/case, per sample or per analysis) and categorise resources differently (e.g., pre-sequencing, sequencing, post-sequencing versus labour, equipment, consumables, storage, bioinformatic analysis). Studies have been inconsistent with the inclusion of costs for capital equipment, labour and analysis while others have not justified the scope of resources [25]. These different approaches for the same technologies, hamper our understanding of key cost drivers [26]. The precision and validity of all cost inputs for a new technology is likely to be important for policy decisions where the accuracy and scale-up could influence resource allocation decisions at a population level.

In light of the variation in cost-analysis reporting and due to the limited number of studies available, we undertook a cost-analysis on genomic sequencing currently occurring for patients with cancer. By reporting costs in a more standardised way, the aim of our analysis was to compare costs of sequencing across different genomic technologies and identify the main drivers of per-patient costs.

## Methods

### Approach & cost perspective

Six genomic sequencing cancer applications were selected for this analysis, based on data availability. Selected applications were comprised of targeted panel, exome and whole genome sequencing technologies. Using six applications of genomic sequencing and different cancer types helps to assess the variability by cancer type. We employed a combination of gross costing (aggregated resource use for a group and separated into per patient units) with micro-costing (units per patient) and valued using a bottom-up approach (i.e., costs assigned per unit) [27]. Micro-costing provides a detailed, reliable and accurate approach for directly measuring activities and resources. Costs were assigned to the unit-level of resources used and aggregated. Even though some resources are fixed, for example employing staff to work in a laboratory over a fixed period, the units involved such as hours of staff time can vary and were apportioned to the time spent on the patient samples. We took the health provider’s perspective (i.e., state government hospital) because genomic services will become routine practice within the government-funded health service. Public hospital care is provided free to Australian citizens under Medicare arrangements. A societal perspective including all costs to third-payers (e.g. insurers, patients) may have resulted in higher sequencing costs. We followed costing methods by Drummond (2005, 28).

### Rationale for inclusion & exclusion of resource types

**Fig. 1:**
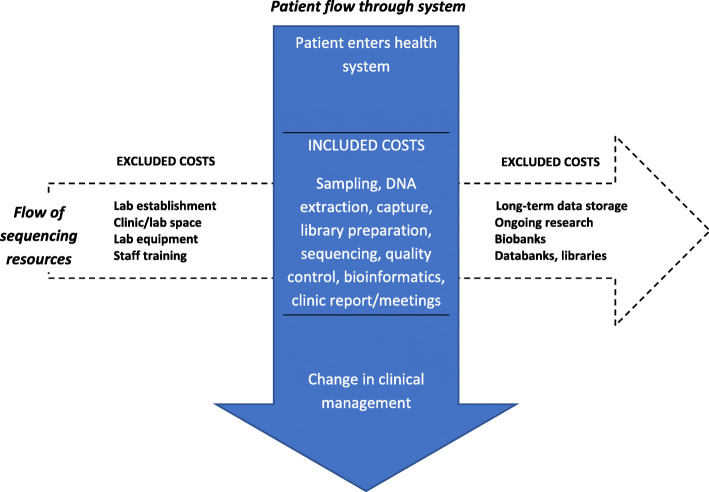
Diagram of the scope of resources included in the cost-analysis

A cost-analysis has three steps: 1) identifying the resources used; 2) measuring, counting or allocating them to units of output and; 3) applying monetary estimates. Included in this analysis were the costs of obtaining the tissue sample through to providing the test information to the clinician (Fig. 1). These include resources for obtaining the sample, laboratory staff time (and overheads), consumables, analysis, data storage and report generation and providing results to clinicians. We included resources necessary for routine sequencing, focusing on resources relevant to practice [10] including sampling or extraction errors. We included sequencing instrumentation and maintenance separately because capital equipment would not normally be covered in a government health service operating budget. The costs of obtaining tumour samples were not included because tumours are biopsied and stored in the normal course of diagnosis. We excluded other infrastructure required to set up a sequencing laboratory such as general equipment (e.g., benchtops, fridges), staff training and national laboratory accreditations. We excluded ancillary resources, defined as long-term genomic data storage (greater than 5 years), ongoing research discovery, biobanks and databanks. Despite these ancillary resources being useful to advance scientific knowledge and enhance future clinical decisions, they are outside the remit of the typical hospital system operating costs [28]. Indeed, in many jurisdictions, research activities are ineligible for public funding through the healthcare system. In Australia, for example, consideration of Medicare reimbursement of tests or procedures on the Medicare Benefits Scheme cannot involve research activity [28].

### Data sources

Data were obtained for six projects involving genomic sequencing for patients with cancer in Brisbane, Australia from June 2017 to June 2018. The projects included a total of 1433 patients with melanoma (383 saliva samples), melanoma or lung cancer (745 tumour samples), oesophageal cancer (100 blood/tumour matched samples), lung cancer (10 blood tumour matched samples) breast cancer (192 blood samples) or mesothelioma (3 blood/tumour matched samples). For each setting, we collected data from laboratory staff, project records and public hospital databases (Additional file 1 for full details). We audited the type and quantity of resources in each project and the associated prices. Market prices for laboratory supplies were provided by each project staff or estimated from the market. Labour costs were valued using a common wage scale with hourly rates of scientific laboratory staff at either AU$46.57 or AU$51.86, including 20% staff overheads, depending on the seniority of the task.

### Documenting testing specifications

Baseline testing specifications were tabulated where relevant, and features known to influence the cost of genomic sequencing in clinical settings were considered. These were the: purpose of testing, sequencing method, sequencing platforms, reading depth/coverage, automation, extent of checking, validation and confirmatory testing, supplier prices, services outsourced, bioinformatics experience and data storage (refer to Additional file 2 for more details).

### Sorting resources and costs into categories

Resource quantities and costs were categorised into seven broad steps representing the logical flow of activities for genomic sequencing. These steps include: 1) sampling, 2) DNA extraction, 3) library preparation, 4) sequencing, 5) analysis, 6) data storage and; 7) reporting to clinicians. Generation of a final report summarising the results of sequencing was included in step 5 ‘analysis’. We estimated short-term storage of sequencing data (i.e., 5 year – given cloud storage is potentially very long term) on the basis of ‘near line’ or infrequent use of cloud storage at USD 0.01 per gigabyte (GB) and we tested for ‘regional’ or frequent use at USD 0.025 per GB and ‘coldline’ or very infrequent use USD 0.007 per GB in sensitivity analysis. Due to library preparation and sequencing steps being outsourced to commercial providers in three projects, no breakdown of these costs were possible and therefore, steps 3 and 4 were combined. Library preparation and sequencing costs of the lung/melanoma project were combined.

To detect insertions, deletions and large genomic rearrangements that are strongly associated with hereditary breast cancer, multiplex ligation-dependent probe amplification was required as an additional step in the breast cancer project. This was included in step 4 ‘sequencing’. It was not required for the lung/melanoma cancer project because they did not aim to detect these types of genetic variants and the other projects involved exome/genome sequencing technologies which cover these variants.

### Analyses

All analyses were undertaken in Excel™. The main unit of analysis was ‘per patient’ as opposed to per sample where matched blood and tumour samples are required per case. Costs were assigned and aggregated for each project by the seven broad categories. Costs were also aggregated according to labour, consumables, testing, equipment and ‘other’ categories. One-way sensitivity analyses on items with known variation or were provided in ranges (e.g., estimates of staff salaries and data storage costs) were performed and tornado diagrams produced. For capital equipment, we obtained the annual equivalent cost calculated by the estimated sequencer acquisition divided by an annuity factor and accounting for useful life (5 years) and interest rate (3%) [29]. This annual cost was divided by the estimated samples per patient throughout 1 year. The annual equivalent cost inputs were tested in sensitivity analyses. Since resource use and costs were deterministic, it was not possible to perform bootstrapping to provide patient-level variation. Costs were reported in 2018 Australian dollars (AUD) and USD (1 USD = AUD 0.6929, www.xe.com).

## Results

Details of the projects’ baseline sequencing specifications are provided in Table 1 and full details of resource types and quantities in Additional file 1 . There were few commonalities across the six projects as they differed in sample type (blood, saliva, tumour tissue), sequencing platform/instrumentation (e.g., Illumina Xten™, NextSeq™, MiSeq™, BGISEQ-500™ [30]), and quality control processes. In addition, three projects sequenced both tumour and blood DNA to allow identification of tumour-specific pathogenic variations. Consequently, the resources used, and their associated costs varied between projects and within sequencing methods (Table 2).

**Table 1:** Description of cancer projects for assessing costs of genomic sequencing

	**Lung/Melanoma cancer**	**Breast cancer**	**Melanoma**	**Lung cancer**	**Oesophageal cancer**	**Mesothelioma**
**Description**						
Project goal	Direct clinical application: treatment pathway	Direct clinical application: treatment pathway	Risk stratification, surveillance decisions	Determine personalised treatment paths	Determine tumour specific mutations	Determine tumour specific mutations
Setting	Statewide health service	Statewide health service	Clinical research	Clinical research	Research	Research
Type of sequencing	Panel	Panel	Exome	Exome	Genome	Genome
Number of patients	745	192	383	10	100	3
Commercial services	No, in-house	No, in-house	Yes, freight/sequencing	No, in-house	Yes, freight/sequencing	Yes, freight/sequencing
Location of sequencing testing	Pathology Queensland, Brisbane	Pathology Queensland, Brisbane	Australian Genomic Research Facility, Melbourne	Australian Translational Genomics Centre, Brisbane	Kinghorn Centre Clinical Genomics, Melbourne	Beijing Genomics Institute, Hong Kong
Setting/Location of bioinformatics	State government health service, Brisbane	State government health service, Brisbane	University, Brisbane	University, Brisbane	Medical Research Institute, Brisbane	Medical Research Institute, Brisbane
**Steps**						
1.Sampling	Biopsy sample (no blood)	Blood sample (no tumour)	Saliva - Oragene DNA self-collection kit	Biopsy sample & blood draw	Tumour biopsy & blood draw	Tumour biopsy & blood draw
2.DNA extraction	DNA Investigator extraction kit, Qubit dsDNA Broad Range Assay kit	QiaSymphony kit, Qubit dsDNA Broad Range Assay kit	QiaQuick Gel Extraction Kit, Qubit dsDNA Broad Range Assay kit, Sangar seq validation, BigDye sequencing kit	QiaSymphony kit, DNA midi kits (blood and tumour)	Qiagen AllPrep DNA mini kit, QiaAMP DNA blood mini kit, Qubit dsDNA Broad Range Assay kit	Qiagen AllPrep DNA/RNA/ miRNA Universal kit, Qubit, SNP arrays
3.Library preparation	Standardised in-house protocol	Standardised in-house protocol	AGRF - Illumina protocols, automated electrophoresis & qPCR	Pre-sequencing qPCR	GenomeOne – Illumina protocols	MGIEasy™ DNA Library Prep Kit V1, DNA nanoballs on BGISEQ-500
4.Sequencing	Illumina MiSeq™/ NextSeq™ TruSeq 26-gene panel	Illumina MiSeq™/ NextSeq™,In-house panel, MLPA	Illumina NovaSeq™	Illumina NextSeq™	Illumina HiSeq XTen™	BGISEQ-500™
Coverage depth	1000X	500X	>100X	100-130X exome, 500-700X spiked-in gene panel	30X blood, 60X tumour	28X blood, 50X tumour
5. Analysis	In-house	In-house	In-house	Demultiplexed CASAVA	In-house	In-house
Software for read mapping, variant calling/ annotation	VariantStudio	VariantStudio Next Gene Soft Genetics	BWA alignment, Picard, GATK Haplotype Caller. ANNOVAR	Novalign, GATK Haplotype Caller, VEP	SNPs, Dual caller qSNP and GATK Haplotype caller. Indels:Haplotype Caller, Structural rearrangements: qSV Copy Number: ascatNGS	SNPs, Dual caller qSNP and GATK Haplotype caller. Indels:Haplotype Caller, Structural rearrangements: qSV Copy Number: ascatNGS
6. Reporting to clinicians	Standard report – paper and electronic, multidiscip team meeting 10% cases	Standard report – paper and electronic, multidisciplinary team meeting 10% cases	n/a pre-clinical work	Prep time for multidisciplinary team	n/a pre-clinical work	n/a pre-clinical work
7. Storage needs, 5 yrs	107 TB (2 GB/sample)	28 TB (2 GB/sample)	275 TB (10 GB/sample)	29 TB (20 GB/sample)	529 TB (150 GB/tumour, 72 GB/blood)	26 TB (150 GB/tumour, 72 GB/blood)

Abbreviations: *DNA* deoxyribose nucleic acid, *GAKT* Genome Analysis Toolkit, *MLPA* Multiplex ligation-dependent probe amplification, *qPCR* quantitative polymerase chain reaction, *BWA* Burrow-Wheeler Aligner, *VEP* Variant Effect Predictor

**Table 2:** Summary of per person costs of genomic sequencing (AU$)

	**Lung cancer/melanoma**	**Breast cancer**	**Melanoma**	**Lung cancer**	**Oesophageal cancer**	**Mesothelioma**
No. of patients	745	192	383	10	100	3
Description	tumour samples panel	blood samples panel	saliva samples exome	blood/tumour pairs exome	blood/tumour pairs genome	blood/tumour pairs genome
1.Sampling						
-saliva/blood^a^	$0.00	$25.05	$27.50	$25.05	$25.05	$25.05
% of total	0.0%	7.2%	3.2%	0.9%	0.5%	0.9%
2.DNA extraction						
-consumables^b^	$22.13	$39.37	$19.28	$39.99	$84.19	$72.69
-validation / quality control	$5.34	$5.34	$32.91	$81.50	$180.70	$139.90
-labour	$0.78	$0.78	$13.86	$63.86	$35.39	$31.05
% of total	6.6%	13.1%	7.6%	6.6%	6.2%	8.4%
3.Library preparation & Capture						
-consumables^c^	$322.23	$149.67	Included in sequencing	$1050.21	Included in sequencing	Included in sequencing
-pre-sequencing quality control	$0.78		n/a	$11.54	n/a	n/a
-labour	$6.31	$6.79	n/a	$139.72	n/a	n/a
% of total	76.8%	45.1%		43.1%		
4.Sequencing						
-testing	n/a	$79.67	$750.00	$1111.75	$4188.60	$1631.26^d^
-labour	n/a	$3.88	Included above	$69.86	Included above	Included above
% of total	0.0%	24.1%	86.1%	42.4%	86.7%	56.3%
5. Analysis						
-computing software	$1.74	$3.20	n/a	$0.73	n/a	n/a
-labour	$59.74	$23.82	$17.29	$57.63	$98.54	$777.96
% of total	14.3%	7.8%	2.0%	2.1%	2.0%	26.9%
6. Storage						
-data storage	$1.96	$1.96	$9.79	$39.15	$217.28	$217.28
% of total	0.5%	0.6%	1.1%	1.4%	4.5%	7.5%
7. Reporting to clinicians						
-multidisciplinary team meeting	$7.56	$7.56	n/a (project is pre-clinical)	$96.54	n/a (project is pre-clinical)	n/a (project is pre-clinical)
% of total	1.8%	2.2%				
**TOTAL per person**	**AU$428.56 (US$240)**	**AU$347.08 (US$297)**	**AU$870.63 (US$604)**	**$2787.53 (US$1932)**	**AU$4829.76 (US$3447)**	**AU$2895.19 (US$2006)**
Capital costs						
-sequencing machine	$22.46	$22.46	$61.58	$131.01	$62.45	$74.24
-maintenance	$11.23	$11.23	$30.79	$65.51	$31.22	$37.12
Total	$33.69	$33.69	$92.36	$196.52	$93.67	$111.36
% extra from total per person	7.9%	9.7%	10.6%	7.0%	1.9%	3.8%
TOTAL per person including capital	$462.25	$380.77	$963.00	$2984.07	$4923.53	$3006.65

^a^The costs of tumour samples were not included because these were routinely biopsied in the normal course of diagnosis and not specifically for sequencing

^b^Includes tube racks, plate racks, storage racks, tips, tubes, ethanol, tube & lid cap strips, sealing film, DNA gel stain, gel extraction kit, microplate buffer etc. (see Additional file 1 for details)

^c^Includes reagents, tips, beads, prep kit, microtubes, plates, PCR plates, TapeStation Assay (see Additional file 1 for details)

^d^USD 600 converted to AUD xe.com, 1.29038 exchange rate

Per patient costs were AU$871 for melanoma (exome sequencing), AU$2788 for lung cancer (exome sequencing), AU$4830 for oesophageal cancer (genome sequencing), AU$429 for lung cancer/melanoma (targeted panel), AU$347 for breast cancer (targeted panel) and AU$2895 for mesothelioma (genome sequencing) (Table 2). Additional capital costs for sequencers ranged from AU$34–197 (2018 €21–61). There were large cost differences within the same technology (i.e., panel, exome, genome). Variations in the melanoma and lung cancer exome sequencing costs were due to both tumour and blood sampling required for lung cancer (relating to different purposes) and to bulk pricing applied for outsourced sequencing in the melanoma project. Costs for consumables accounted for the main differences within targeted panels.

As a proportion of total per patient costs (excluding capital equipment), three-quarters were for the combined library preparation and sequencing steps (average 76.8%, range 56.3 to 86.7%), while the other steps were small by comparison; DNA extraction (average 8.1%, range 6.2 to 13.1%), data analysis (average 9.2%, range 2.0 to 26.9%) and data storage (average 2.8%, range 0.5 to 7.5%) (Table 2). The costs of capital equipment and maintenance represented 1.9–10.6% additional costs. Consumables for library preparation, capture and sequencing were the most expensive cost steps (Table 3) that were outsourced to commercial providers in three projects. Where labour costs were explicit for all steps and not embedded in ‘sequencing’ by external providers (i.e., lung/melanoma, breast and lung cancer projects), the percentage of costs ranged from 11.2 to 16.1%, including bioinformatic analyses (Table 3).

**Table 3:** Per person costs of genomic sequencing by types of resources (AU$)

**Totals by types of costs**	**Lung cancer/melanoma**	**Breast cancer**	**Melanoma**	**Lung cancer**	**Oesophageal cancer**	**Mesothelioma**
	**Panel**	**Panel**	**Exome**	**Exome**	**Genome**	**Genome**
- labour	$74.38	$42.83	$31.15	$427.60	$133.94	$809.01
- consumables^a^	$344.36	$189.04	$19.28	$1090.21	$84.19	$72.69
- testing^b^	$6.12	$85.01	$782.91	$1204.79	$4369.30	$1771.16
- equipment^c^	$35.43	$36.88	$92.36	$197.25	$93.67	$111.36
- other^d^	$1.96	$27.01	$37.29	$64.22	$242.43	$242.43
Total	**AU$462.25** **(US$320)**	**AU$380.77** **(US$264)**	**AU$963.00** **(US$667)**	**AU$2984.07** **(US$2068)**	**AU$4923.53** **(US$3412)**	**AU$3006.65** **(US$2084)**
**% of total by types of costs**						
- labour	16.1%	11.2%	3.2%	14.3%	2.7%	26.9%
- consumables	74.5%	49.6%	2.0%	36.5%	1.7%	2.4%
- testing	1.3%	22.3%	81.3%	40.4%	88.7%	58.9%
- equipment	7.7%	9.7%	9.6%	6.6%	1.9%	3.7%
- other	0.4%	7.1%	3.9%	2.2%	4.9%	8.1%
**Total %**	**100.0%**	**100.0%**	**100.0%**	**100.0%**	**100.0%**	**100.0%**

^a^Consumables for Melanoma, Oesophageal cancers & Mesothelioma are included in ‘testing’ as these were outsourced to commercial services and could not be separately identified

^b^Testing includes validation, pre-sequencing testing and outsourced sequencing

^c^Equipment includes maintenance costs

^d^Other includes sampling, data storage, software, multidisciplinary team meetin

**Fig. 2:**
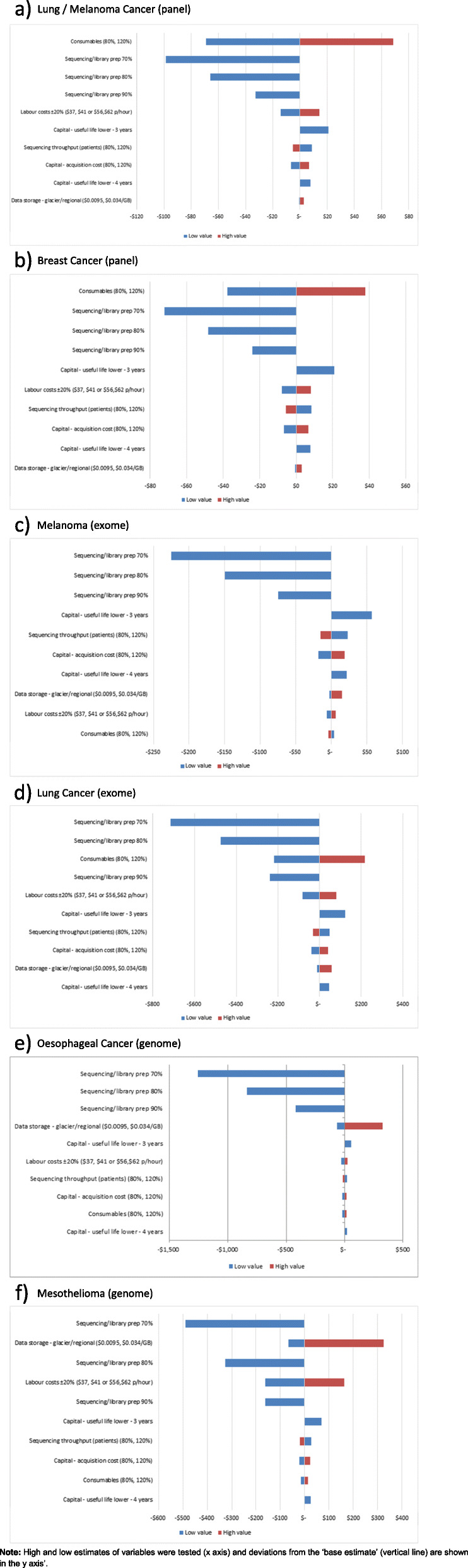
**a**-**d**. Sensitivity analyses

Sensitivity analyses were performed on several variables where values were uncertain (Additional file 2 and Additional file 3 ). Tornado diagrams are presented for the six cancer projects (Fig. 2a-f). Across all projects, reductions in the costs of the combined steps of library preparation and sequencing by 10, 20 and 30%, had the greatest change in the total costs across the applications, decreasing by 7.2, 14.4 and 21.6%, respectively. By contrast, other components were less important in driving costs. When the salaries of scientists performing DNA extraction, sequencing and analysis were 20% lower or higher than the pay level used (as per current health department pay scales), the total costs varied by 2.4% (range 0.5 to 5.4%) across the six projects. When data storage costs were increased to USD 0.025 per GB per month (regional use), the total costs were up to 0.8% higher for targeted panels, ~ 2.0% higher for exome sequencing projects but were 6.6 to 10.9% higher for whole genome sequencing projects.

## Discussion

Resources used in the workflow of genomic sequencing are complex and reflect many processes. Our study presents the range of operating cost-outlays across active projects for cancer genomics in Brisbane, Australia. Although it is well known that whole genome sequencing costs more than exomes, and exome sequencing costs more than targeted panels, here we show that there is variation in costs within the same technologies. In this study, they varied by AU$81 per patient between the targeted panels (sequencing only one sample/patient) to AU$1917 within exome, and AU$1935 within whole genome sequencing methods. Our findings suggest that the purpose and extent of sequencing (whether tumour and matched blood DNA samples were needed) along with the commercial prices offered were the main points of difference across the projects. Future work is needed to assess costs by different cancer types as we did not have sufficient cases and cancer types here to inform this. Documenting baseline sequencing specifications and categorising resources into seven logical steps aided comparisons and could be used by others in future work. As there are many factors that determine the resources and costs involved in sequencing, projects need to be sufficiently detailed to ensure operating costs are captured to allow for informed assessments.

Several others have published studies on micro-costing approaches for genomic sequencing [13, 17, 19, 23, 24]. Published since 2016, mean per patient costs for sequencing (and not subsequent management) vary from CA$1029 (2018 US$803) [19], US$699–1949 (2018 US$741–2067) [17] and €1669 (2018 US$1531) [23] for panel testing, US$1499–$2428 (2018 US$1590-2575) [17] and CA$1655 (2018 US$1292) [14] for exome sequencing and €3858 (2018 US$3502) [24] and CA$34,886 (2018 US$27,227) [13] for genome sequencing. Our findings show similar costs by these three sequencing technologies, although were in the lower range (including capital costs). Similar to van Nimwegen et al. (2016) where capital costs were 0.6–10.5% of total costs by panel testing up to whole genome testing, our findings found the range was 1.9–10.6% using conservative caseloads [23]. While studies differ by cost components included and the descriptions of sequencing activities, a common finding is that consumables and sequencing materials are the key drivers in costs [3, 16, 23], and reductions in these will have the greatest impact on overall sequencing costs in future [23]. Our study used the patient as the unit of analysis, rather than per sample [23], to align with economic evaluations of new technologies that typically assess patient disease journeys.

In practice, there are instances where the quality of the DNA from the sample fails to meet the high standards required for genomic sequencing. The resulting need for sample retrieval and re-sequencing, impacts on resource use and costs. For example, 30% of the oesophageal cancer samples did not have the minimum 40% tumour content required so another tumour biopsy was retrieved (from three tumour biopsy samples per patient originally stored). Since this is typical, costs were appropriately included for the DNA to be re-extracted and quality control re-done. However, better biopsy samples present an opportunity for cost savings. For the melanoma project, there were no instances of saliva samples having insufficient DNA to perform the extraction however the quality of some formalin-fixed paraffin-embedded material was poor and 30 samples had to be prepared again, re-sent to the sequencing laboratory and re-sequenced. This incurs extra costs for the commercial provider but did not affect their fee charged.

The two whole genome applications, mesothelioma and oesophageal cancer, were pre-clinical while melanoma and lung cancer applications were clinical demonstration projects using exome sequencing. The lung/melanoma and breast cancer panel applications are routinely implemented within the health system. Four of the six projects involved research which presents difficulties in quantifying and valuing resources that need research and clinical activities to be separated. Separating research from clinical sequencing activity may be problematic for genomic technologies when researchers are closely involved in the bioinformatics stages and are discovering new important variants for clinical attention. While the social value of genomic research may be significant, it is likely to remain difficult to measure and value. Gene discovery is rapidly evolving and could be viewed as a necessary consideration for costing with re-analysis of stored data likely to be more commonplace and will give rise to higher costs per patient than presented here.

Our findings improve the understanding of the many cost components of genomic sequencing, highlight the difficulties in measuring costs in a highly commercialized area and should assist health economists who are undertaking cost-effectiveness analyses of clinical genomics. Costs and cost proportions (both in terms of cost categorization and cost type) were compared across and within technologies which provided some stark and interesting contrasts. It should be emphasized that the costs presented here reflect the commercial prices involved in sequencing activities where there is limited market competition. The bulk pricing of the melanoma setting, where 383 salvia samples were tested in one batch, may be closer to the actual cost of library preparation and sequencing. While in the lung cancer setting with only 10 patients, there were very detailed quantities of resource use for exome sequencing. It is perhaps a combination of these two settings that provide a reliable indication of the cost of exome sequencing and may be useful for budget impact analyses. Ultimately, in the context of a cost-effectiveness analysis, the value of genomic sequencing is the goal for decision makers and capturing the implications on patient management is critical.

This study has some limitations. Virtually all the monetary values reported are ‘prices’ rather than true costs because there is no other practical way of valuing them. While we have assumed prices are a reasonable proxy for costs, with competition in the market increasing, these prices are likely to still overestimate true costs because they include mark-ups and other fees. There are significant disparities between the number of patients within the exome sequencing estimates (melanoma and lung cancer) and whole genome sequencing (oesophageal cancer and mesothelioma) and caution is required when comparing between these technologies. Some monetary costs were difficult to estimate as hospital or laboratory records did not have these readily available (e.g., costs of data storage and tissue sampling), or laboratory funding was not organised on a per project basis for straight-forward calculation. For example, in one setting scientists are allocated laboratory consumables over a set term irrespective of caseload and specific projects. Furthermore, costs of data storage were valued from online cloud-based pricing, but the required storage duration is unclear, and prices are determined by how often the data would need to be re-analysed, if at all. The estimation of capital equipment is problematic with sequencing costs since new systems are being developed every other year and estimating useful life for amortization may be overestimated (which underestimates costs). Our sensitivity analyses showed different inputs to calculating capital equipment had a minor impact on total costs. Genomic medicine in Brisbane is emerging and further capacity for full implementation is required. It is likely the costs may change as projects use local sequencing systems, workforce capacity grows, and sequencing becomes more streamlined. Finally, we were not able to assess marginal costs or variation across individuals via bootstrapping methods as data was not collected at the individual level but rather was deterministic. Ideally, a stronger design would capture individual-level data that could be statistically analysed, although it may still be challenging to obtain accurate per patient resource units.

Claims of sequencing now dropping to US$1000 per genome have arisen with more powerful sequencing platforms but there is little evidence to support this claim [16]. It is unclear how sequencing costs will evolve in the future, but large drops are probably unlikely due to fixed resource components and more complex bioinformatic analyses required [23]. With emerging sequencing methods and automated processes, these cost drivers will continue to fluctuate until more standardized, steady state sequencing methods are developed. In future, researchers should fully report the costs involved in sequencing, as per the seven components here, to enable trends to be more accurately monitored.

## Conclusion

Genomic sequencing costs within cancer vary with the purpose of testing such as risk prediction or personalised treatment (dictating the need for germline only or matched germline and tumour samples per patient), and the commercial sequencing rates offered, among others. Using the natural steps involved in sequencing and categorising resources accordingly, future evaluations of sequencing costs could be more standardised and facilitate easier comparison of cost drivers. Future work assessing individual-level costs for the same sequencing approach by different cancer types are required.

## Supplementary information

**Additional file 1.** Full details of costing estimates.

**Additional file 2.** Features of sequencing that will influence costs.

**Additional file 3.** Calculation of annual per person cost of sequencers.

## Data Availability

The datasets used and/or analysed during the current study are provided in the Supplementary files and available from the corresponding author on reasonable request.

## Abbreviations

AUD: Australian dollars
BWA: Burrow-Wheeler Aligner
CA: Canadian dollars
DNA: Deoxyribose nucleic acid
GAKT: Genome Analysis Toolkit
GB: Gigabytes
MLPA: Multiplex ligation-dependent probe amplification
qPCR: Quantitative polymerase chain reaction
USD: United States dollars
VEP: Variant Effect Predictor
