# Supplementary material for: Estimating the costs of genomic sequencing in cancer control
Source: BMC Health Serv Res. 2020 Jun 3;20:492. doi: 10.1186/s12913-020-05318-y (PMC7268398; doi:10.1186/s12913-020-05318-y)
Supplement: Supplementary file 1 — Additional file 1. Full details of costing estimates. [file 12913_2020_5318_MOESM1_ESM.docx]

**APPENDIX 1: FULL DETAILS OF COSTING ESTIMATES**

1. Lung/Melanoma Project (tumour sample) n =745

| **Description** | **Company** | **Catalog Number** | **Units** | **Unit Price** | **Total price** | **Samples per unit** | **Price/sample** | **Price per project** |
| --- | --- | --- | --- | --- | --- | --- | --- | --- |
| **1.Sampling** | |  |  |  |  |  |  |  |
| -biopsy sample, no additional costs |  |  |  |  |  |  | $ - |  |
|  |  |  |  |  |  |  | Subtotal | **$ -** |
| **2.DNA extraction** | |  |  |  |  |  |  |  |
| DNA Investigator extraction kit (1 box of 50) |  |  | 1.00 | $ 405.60 | $ 405.60 | 50 | $ 8.11 |  |
| Tips 10uL | Eppendorf | 30077504 | 1.00 | $ 192.00 | $ 192.00 | 96 | $ 4.00 |  |
| Tips 20uL | Eppendorf | 30077539 | 1.00 | $ 183.60 | $ 183.60 | 96 | $ 1.91 |  |
| Tips 200uL | Eppendorf | 30077555 | 1.00 | $ 190.80 | $ 190.80 | 96 | $ 7.95 |  |
| Tips 1000uL | Eppendorf | 30077857 | 1.00 | $ 205.00 | $ 205.00 | 96 | $ - |  |
| 96-well Tube Storage Racks |  |  | 1.00 | $ 6.90 | $ 6.90 | 96 | $ 0.07 |  |
| 8-well strip |  |  | 4.00 | $ 1.03 | $ 4.12 | 50 | $ 0.02 |  |
| 96 well plate |  |  | 1.00 | $ 3.36 | $ 3.36 | 50 | $ 0.07 |  |
| -total consumables |  |  |  |  |  |  | $ 22.13 | $ 16,494.45 |
| Scientist - 2.5 hours per batch | QP |  | 2.50 | $ 46.57 | $ 116.43 | 24 | $ 4.85 |  |
| Scientist - 0.25 hours per batch |  |  | 0.25 | $ 46.57 | $ 11.64 | 24 | $ 0.49 |  |
| -labour / processing |  |  |  |  |  |  | $ 5.34 | $ 3,976.67 |
| Qubit dsDNA Broad Range Assay Kit | Invitrogen | Q32853 | 1.00 | $ 389.72 | $ 389.72 | 500 | $ 0.78 |  |
| -validation / QC |  |  |  |  |  |  | $ 0.78 | $ 580.84 |
|  |  |  |  |  |  |  | Subtotal | **$ 21,051.95** |
| **3.Library preparation** | |  |  |  |  |  |  |  |
| TruSight Tumour 26-gene panel kit |  |  | 1.00 | $ 5,190.64 | $ 5,190.64 | 24 | $ 216.28 |  |
| HYP_Plate |  |  |  |  |  |  |  |  |
| 96 well-skirted PCR plate, 0.2ml polypropylene | Bio-Rad | MSP-9601 | 1.00 | $ 2.20 | $ 2.20 | 24 | $ 0.09 |  |
| Adhesive aluminimum foil seal | Beckman Coluter | 538619 | 1.00 | $ 1.67 | $ 1.67 | 24 | $ 0.07 |  |
| Nuclease-Free Water (10 x 50 ml) | Qiagen | 129114 | 1.00 | $ 119.00 | $ 119.00 | 100 | $ 1.19 |  |
| DNA Hydration Solution (100 ml) (Sodium Hydroxide solution) | Sigma | 158914 | 1.00 | $ 126.00 | $ 126.00 | 400 | $ 0.32 |  |
| 96-well plate |  |  | 1.00 | $ 3.36 | $ 3.36 | 24 | $ 0.14 |  |
| Ethanol | Chem-Supply | EA043-2.5L | 1.00 | $20 | $20 | 2000 | $ 0.01 |  |
| MiSeq Reagent Kit v3, 600 Cycles | Illumina |  | 2.00 | $ 1,247.97 | $ 2,495.94 | 24 | $ 104.00 |  |
| QC Phix Control Kit - need 3 per year right now |  |  | 1.00 | $ 135.66 | $ 135.66 | 994 | $ 0.14 |  |
| -consumables^1^ |  |  |  |  |  |  | $ 322.23 | $ 240,123.53 |
| Qubit dsDNA Broad Range Assay Kit | Invitrogen | Q32853 | 1.00 | $ 389.72 | $ 389.72 | 500 | $ 0.78 |  |
| -pre-sequencing QC |  |  |  |  |  |  | $ 0.78 | $ 580.84 |
| Scientist - 0.75 hours per batch |  |  | 0.75 | $ 46.57 | $ 34.93 | 24 | $ 1.46 |  |
| Scientist - 2.5 hours per batch |  |  | 2.50 | $ 46.57 | $ 116.43 | 24 | $ 4.85 |  |
| -labour |  |  |  |  |  |  | $ 6.31 | $ 4,699.70 |
|  |  |  |  |  |  |  | Subtotal | **$ 245,404.07** |
| **4.Sequencing** | |  |  |  |  |  |  |  |
|  |  |  |  |  |  |  |  |  |
|  |  |  |  |  |  |  |  |  |
| **5. Analysis** | |  |  |  |  |  |  |  |
| VariantStudio licence |  | (Illumina) | 1.00 | 6000 | $ 6,000.00 | 3442 | $ 1.74 |  |
| -software/IT |  |  |  |  |  |  | $ 1.74 | $ 1,299.09 |
| VariantStudio output into worksheet |  |  | 1.00 | $ 46.57 | $ 46.57 | 1 | $ 46.57 |  |
| Checking EGFR samples - IGB |  |  | 0.50 | $ 46.57 | $ 23.29 | 24 | $ 0.97 |  |
| Analysis and reporting- operator |  |  | 3.50 | $ 46.57 | $ 163.00 | 24 | $ 6.79 |  |
| Analysis and reporting - validator |  |  | 2.50 | $ 51.86 | $ 129.66 | 24 | $ 5.40 |  |
| -informatics labour |  |  |  |  |  |  | $ 59.74 | $ 44,515.64 |
|  |  |  |  |  |  |  | Subtotal | **$ 45,814.73** |
| **6. Storage** |  |  |  |  |  |  |  |  |
| - cloud storage, near line per GB AU$0.0136 per month |  | 5 years | 1490 | $1,459.40 | $1,459.40 |  | $ 1.96 | **$ 1,459.40** |
| - 2 GB per sample, 5 years |  |  | 107308.80 |  |  |  |  |  |
| **7. Reporting to clinicians** | |  |  |  |  |  |  |  |
| -generating report | above |  |  |  |  |  |  |  |
| -multidisciplinary care team meeting prep |  |  |  | 75.58 |  |  | $ 7.56 | **$ 5,632.22** |
|  |  |  |  |  |  |  |  |  |
|  |  |  |  |  |  | **TOTAL** | | **$ 319,362.37** |
|  |  |  |  |  |  | **TOTAL per person** | | **$ 428.56** |
|  |  |  |  |  |  |  |  |  |
| **8. Capital - sequencing machine** |  |  |  |  |  |  |  |  |
| NextSeq 500 | Illumina | Acquisition cost | $ 360,000.00 | Throughput (pts) | 3500 |  |  | $ 22.46 |
| Maintenance costs | 50% of capital cost per patient | |  |  |  |  |  | $ 11.23 |
|  |  |  |  |  |  |  |  | **$ 33.69** |
|  |  |  |  |  |  | **TOTAL with capital** |  | **$ 451.02** |

1. Breast Cancer Project (blood sample) n =192

| **Description** | **Company** | **Catalog Number** | **Units** | **Unit Price** | **Total price** | **Samples per unit** | **Price/sample** | **Price per project** |
| --- | --- | --- | --- | --- | --- | --- | --- | --- |
| **1.Sampling** | |  |  |  |  |  |  |  |
| -blood sample |  | Medicare Benefit Scheme#13839 | 1.00 | $ 23.05 | $ 23.05 | 1 | $ 23.05 |  |
| -courier/freight to lab |  |  |  | $ 2.00 | $ 2.00 | 1 | $ 2.00 |  |
|  |  |  |  |  |  |  | $ 25.05 |  |
|  |  |  |  |  |  |  | Subtotal | **$ 4,809.60** |
| **2.DNA extraction** |  |  |  |  |  |  |  |  |
| QIAsymphony kit |  |  | 1.00 | $ 814.10 | $ 814.10 | 50 | $ 16.28 |  |
| Tips 10uL | Eppendorf | 30077504 | 1.00 | $ 192.00 | $ 192.00 | 96 | $ 4.00 |  |
| Tips 20uL | Eppendorf | 30077539 | 1.00 | $ 183.60 | $ 183.60 | 96 | $ 1.91 |  |
| Tips 200uL | Eppendorf | 30077555 | 1.00 | $ 190.80 | $ 190.80 | 96 | $ 7.95 |  |
| Tips 1000uL | Eppendorf | 30077857 | 1.00 | $ 205.00 | $ 205.00 | 96 | $ 8.54 |  |
| Pipette tips |  |  | 10.00 | $ 6.00 | $ 60.00 | 100 | $ 0.60 |  |
| 8-well strip |  |  | 4.00 | $ 1.03 | $ 4.12 | 50 | $ 0.02 |  |
| 96 well plate |  |  | 1.00 | $ 3.36 | $ 3.36 | 50 | $ 0.07 |  |
| -consumables |  |  |  |  |  |  | $ 39.37 | $ 7,559.82 |
| Scientist - 2.5 hours per batch |  |  | 2.50 | $ 46.57 | $ 116.43 | 24 | $ 4.85 |  |
| Scientist - 0.25 hours per batch |  |  | 0.25 | $ 46.57 | $ 11.64 | 24 | $ 0.49 |  |
| -labour / processing |  |  |  |  |  |  | $ 5.34 | $ 1,024.58 |
| Qubit dsDNA Broad Range Assay Kit | Invitrogen | Q32853 | 1.00 | $ 389.72 | $ 389.72 | 500 | $ 0.78 |  |
| -validation / QC |  |  |  |  |  |  | $ 0.78 | $ 149.65 |
|  |  |  |  |  |  |  | Subtotal | **$ 8,734.05** |
| **3.Library preparation** |  |  |  |  |  |  |  |  |
| AFP2 panel (192)-160 patients |  | FC-130-1009 | 1.00 | $ 20,889.28 | $ 20,889.28 | 160 | $ 130.56 |  |
| TruSeq custom amplicon index kit (384) |  | FC-130-1003 | 0.50 | $ 1,291.20 | $ 645.60 | 160 | $ 4.04 |  |
| custom amplicon filter plate |  | FC-130-1006 | 1.00 | $ 45.00 | $ 45.00 | 24 | $ 1.88 |  |
| Deep well plate |  |  | 3.00 | $ 2.20 | $ 6.60 | 24 | $ 0.28 |  |
| PCR plate |  |  | 4.00 | $ 3.36 | $ 13.46 | 24 | $ 0.56 |  |
| MiSeq reagent micro fixture v2 (300) | Illumina |  | 2.00 | $ 135.66 | $ 271.32 | 24 | $ 11.31 |  |
| QC Phix Control Kit - need 3 per year right now |  |  | 1.00 | $ 135.66 | $ 135.66 | 128 | $ 1.06 |  |
| -consumables |  |  |  |  |  |  | $ 149.67 | $ 28,736.36 |
| Scientist (hours) |  |  | 1.50 | $ 46.57 | $ 69.86 | 24 | $ 2.91 |  |
| Scientist (hours) |  |  | 2.00 | $ 46.57 | $ 93.14 | 24 | $ 3.88 |  |
| -labour |  |  |  |  |  |  | $ 6.79 | $ 1,304.02 |
|  |  |  |  |  |  |  | Subtotal | **$ 30,040.37** |
| **4.Sequencing** |  |  |  |  |  |  |  |  |
| MLPA mix (BRCA1 & BRCA2) |  |  | 1 | $ 2,145.00 | $ 2,145.00 | 36 | $ 59.58 |  |
| plate cover |  |  | 1 | $ 1.67 | $ 1.67 | 24 | $ 0.07 |  |
| PCR plate |  |  | 2 | $ 3.36 | $ 6.73 | 24 | $ 0.28 |  |
| Capillary elextrophoresis |  |  | 1 | $ 760.32 | $ 760.32 | 48 | $ 15.84 |  |
| tips labadvantage p20 |  |  | 2 | $ 183.60 | $ 183.60 | 96 | $ 1.91 |  |
| tips labadvantage p200 |  |  | 1 | $ 190.80 | $ 190.80 | 96 | $ 1.99 |  |
| -consumables^1^ |  |  |  |  |  |  | $ 79.67 | $ 15,297.24 |
| worksheet preparation |  |  | 0.5 | $ 46.57 | $ 23.29 | 36 | $ 0.65 |  |
| qauntification dilution |  |  | 1 | $ 46.57 | $ 46.57 | 36 | $ 1.29 |  |
| hybridisation |  |  | 0.5 | $ 46.57 | $ 23.29 | 36 | $ 0.65 |  |
| extenion ligation |  |  | 0.5 | $ 46.57 | $ 23.29 | 36 | $ 0.65 |  |
| PCR (hours) |  |  | 0.5 | $ 46.57 | $ 23.29 | 36 | $ 0.65 |  |
| -labour |  |  |  |  |  |  | $ 3.88 | $ 745.15 |
|  |  |  |  |  |  |  | Subtotal | **$ 16,042.39** |
| **5. Analysis** |  |  |  |  |  |  |  |  |
| VariantStudio licence | (Illumina) |  | 1 | $ 6,000.00 | $ 6,000.00 | 3442 | $ 1.74 |  |
| Next gene soft genetics |  |  | 1 | $ 5,000.00 | $ 5,000.00 | 3442 | $ 1.45 |  |
| -software/IT |  |  |  |  |  |  | $ 3.20 | $ 613.63 |
| Analysis and reporting- operator (hours) |  |  | 5 | $ 46.57 | $ 232.86 | 24 | $ 9.70 |  |
| Analysis and reporting – validator (hours) |  |  | 4 | $ 51.86 | $ 207.46 | 24 | $ 8.64 |  |
| analysis |  |  | 1 | $ 46.57 | $ 46.57 | 36 | $ 1.29 |  |
| reporting |  |  | 1 | $ 46.57 | $ 46.57 | 36 | $ 1.29 |  |
| analysis |  |  | 1 | $ 51.86 | $ 51.86 | 36 | $ 1.44 |  |
| validation |  |  | 1 | $ 51.86 | $ 51.86 | 36 | $ 1.44 |  |
| -informatics labour |  |  |  |  |  |  | $ 23.82 | $ 4,572.51 |
|  |  |  |  |  |  |  | Subtotal | **$ 5,186.14** |
| **6. Storage** |  |  |  |  |  |  |  |  |
| - cloud storage, near line per GB AU$0.0136 per month |  |  | 384 | $ 376.01 | $ 376.01 |  | $ 1.96 | **$ 376.01** |
|  |  |  | 27648.00 |  |  |  |  |  |
| **7. Reporting to clinicians** | |  |  |  |  |  |  |  |
| -generating report | above |  |  |  |  |  |  |  |
| -multidisciplinary care team meeting prep |  |  |  | $ 75.58 |  |  | $ 7.56 | **$ 1,451.14** |
|  |  |  |  |  |  |  |  |  |
|  |  |  |  |  |  |  |  |  |
|  |  |  |  |  |  | **TOTAL** | | **$ 66,639.71** |
|  |  |  |  |  |  | **TOTAL per person** | | **$ 347.08** |
|  |  |  |  |  |  |  |  |  |
| **8. Capital - sequencing machine** |  |  |  |  |  |  |  |  |
| NextSeq 500 | Illumina | Acquisition cost | $ 360,000.00 | Throughput | 3500 |  |  | $ 22.46 |
| Maintenance costs | 50% of capital cost per patient | |  |  |  |  |  | $ 11.23 |
|  |  |  |  |  |  |  |  | **$ 33.69** |
|  |  |  |  |  |  |  |  |  |
|  |  |  |  |  |  | **TOTAL with capital** | **9.1%** | **$ 369.54** |

1. Melanoma (saliva samples) n=383

| **Description** | **Company** | **Catalog Number** | **Units** | **Unit Price** | **Total price** | **Samples per unit** | **Price/sample** | **Price per project** |
| --- | --- | --- | --- | --- | --- | --- | --- | --- |
|  |  |  |  |  |  |  |  |  |
| **1.Sampling** | | | | | | | | |
| -saliva sample | Oragene DNA self-collection kit |  | 1.00 | $ 27.50 | $ 27.50 | 1.00 | $ 27.50 |  |
| -courier/freight to lab | n/a |  |  |  |  |  | $ - |  |
|  |  |  |  |  |  |  | Subtotal | **$ 10,532.50** |
| **2.DNA extraction** | | | | | | | | |
| PrepIT.L2P (2000 preps) |  | PT-L2P-45 | 1.00 | $ 740.00 | $ 740.00 | 2000 | $ 0.37 |  |
| 96-well Tube Storage Racks | Interpath Services Pty Ltd | 532039 | 1.00 | $ 6.90 | $ 6.90 | 96 | $ 0.07 |  |
| 15/50 ml Tube Rack | Thermo Fisher Scientific Austr | TPP99019 | 1.00 | $ 7.95 | $ 7.95 | 30 | $ 0.27 |  |
| 96-well Plate Rack | Interpath Services Pty Ltd | 523029 | 1.00 | $ 5.90 | $ 5.90 | 96 | $ 0.06 |  |
| SSI 1250uL tips 10rk/pk | LabGear Australia | SSIB4347NSFS | 10.00 | $ 6.00 | $ 60.00 | 100 | $ 0.60 |  |
| SSI 20uL tips 10rk/pk | LabGear Australia | SSIB4237NAFS | 10.00 | $ 5.50 | $ 55.00 | 32 | $ 1.70 |  |
| SSI 200uL tips 10rk/pk | LabGear Australia | SSIB4237NSFS | 10.00 | $ 5.50 | $ 55.00 | 50 | $ 1.10 |  |
| 15 ml Conical Tube | Falcon 15ml Screw Cap Tube pack of 50 | 352096 | 50.00 | $ 7.92 | $ 7.92 | 50 | $ 0.16 |  |
| SAFELOCK MICROTUBE 2ML | VWR International Pty Ltd | EPPE0030120.094 |  | $ 83.79 | $ 83.79 | 524 | $ 0.16 |  |
| Qubit Assay tubes | Thermo Fisher Scientific Austr | Q32856 |  | $ 135.00 | $ 135.00 | 500 | $ 0.27 |  |
| 1.5ml SSI pack of 500 | Interpath Services Pty Ltd | 121000 | 500.00 | $ 17.59 | $ 17.59 | 50 | $ 0.35 |  |
| 200 proof Ethanol Store in Flammable Cabinet | Point of Care Diagnostics Aust | ETHABS95/2.5P |  |  | $ 11.50 |  | $ 0.03 |  |
| Phire Hot Start II DNA Pol | Thermo Fisher Scientific Austr | F122S |  |  | $ 172.00 | 200 | $ 0.86 |  |
| tube+lid flat cap 120strips/pk | Interpath Services Pty Ltd | 324500 |  |  | $ 100.00 | 1000 | $ 0.10 |  |
| Axygen CyclerSeal 60um pk100 (Sealing film Cycle seal) | Fisher Biotec | PCR-TS |  |  | $ 72.00 |  | $ 0.19 |  |
| Oligonucleotides | Sigma-Aldrich Pty Ltd |  |  |  | $ 22.43 |  | $ 0.06 |  |
| Qiaquick Gel Extraction Kit (50) | Qiagen | 28704 |  |  | $ 150.00 | 50 | $ 3.00 |  |
| SYBR Safe DNA Gel Stain | Life Technologies Pty Ltd | S33102 |  |  | $ 127.35 | 161 | $ 0.79 |  |
| 1 Kb Plus DNA Ladder | Life Technologies Pty Ltd | 10787018 |  |  | $ 191.20 | 252 | $ 0.76 |  |
| Agarose I (Molecular Biology Grade) (500g) | Life Technologies Pty Ltd | 17852 |  |  | $ 952.00 | 5011 | $ 0.19 |  |
| MICROPLATE 384WELL 50/PK | Pacific Laboratory Products Pt | PCR-384M2-C |  |  | $ 275.00 | 50 | $ 5.50 |  |
| Tae Buffer 50x (1L) | Thermo Fisher Scientific Austr | 24710030 |  |  | $ 192.00 | 100 | $ 1.92 |  |
| Qubit dsDNA Broad Range Assay Kit | Thermo Fisher Scientific Austr |  | 1.00 | $ 389.72 | $ 389.72 | 500.00 | $ 0.78 |  |
| - consumables |  |  |  |  |  |  | $ 19.28 | $ 7,384.02 |
|  |  |  |  |  |  |  |  |  |
| BigDye Terminator v3.1 Cycle Sequencing Kit (100 reactions) | Thermo Fisher Scientific Austr |  |  | $ 22.91 | $ 2,291.00 |  | $ 22.91 |  |
| AGRF sequence cost (~$10/sample) | AGRF |  |  | $ 10.00 | $ 3,600.00 |  | $ 10.00 |  |
| -validation |  |  |  |  |  |  | $ 32.91 | $ 12,604.53 |
| Scientist - non PhD | 2-3 weeks to prepare samples | 3 weeks | 114.00 | $ 46.57 | $ 5,309.21 | 13.86 | $ 13.86 |  |
| -labour / processing |  |  |  |  |  |  | $ 13.86 | $ 5,309.21 |
|  |  |  |  |  |  |  | Subtotal | **$ 25,297.76** |
| **3.Library preparation** | | | | | | | | |
| Included in AGRF |  |  |  |  |  |  |  |  |
|  |  |  |  |  |  |  |  |  |
| **4.Sequencing** | | | | | | | | |
| AGRF Exome illumina sequencing | AGRF |  |  | $ 750.00 | $ 287,250.00 |  | $ 750.00 |  |
|  |  |  |  |  |  |  | $ 750.00 | **$ 287,250.00** |
| **5. Analysis** | | | | | | | | |
| Total time of 20 minutes per sample | Senior researcher post-doc |  | 127.67 | $ 51.86 | $ 6,621.30 | 6621.30 | $ 17.29 |  |
| -informatics labour |  |  |  |  |  |  | $ 17.29 | $ 6,621.30 |
|  |  |  |  |  |  |  | Subtotal | **$ 6,621.30** |
| **6. Storage** | | | | | | | | |
| - cloud storage, near line per GB AU$0.0136 per month | 22 GB each sample (10 GB BAM, 12 GB FASTQ) for 5 years | 3830 |  | $ 3,750.34 |  |  | $ 9.79 | **$ 3,750.34** |
|  | **Using BAM only** | 275760 |  |  |  |  |  |  |
| **7. Reporting to clinicians** | | | | | | | | |
| -generating report |  |  |  |  |  |  |  |  |
| -multidisciplinary care team meeting prep | NA |  |  |  |  |  | $ - | **$ -** |
|  |  |  |  |  |  |  |  |  |
|  |  |  |  |  |  | **TOTAL** | | **$ 333,451.90** |
|  |  |  |  |  |  | **TOTAL per person** | | **$ 870.63** |
|  |  |  |  |  |  |  |  |  |
| **8. Capital - sequencing machine** | | | | | | | | |
| NovaSeq 500 | Illumina | Acquisition cost | $ 1,410,000.00 | Throughput | 5000 |  |  | $ 61.58 |
| Maintenance costs | 50% of capital cost per patient |  |  |  |  |  |  | $ 30.79 |
|  |  |  |  |  |  |  |  | **$ 92.36** |
|  |  |  |  |  |  |  |  |  |
|  |  |  |  |  |  | **TOTAL with capital** | | **$ 932.21** |

1. Lung Cancer (tumour and blood samples) n=10

| **Description** | **Company** | **Catalog Number** | **Units** | **Unit Price** | **Total price** | **Samples per unit** | **Price/sample** | **Price per project** |
| --- | --- | --- | --- | --- | --- | --- | --- | --- |
|  |  |  |  |  |  |  |  |  |
| **1.Sampling** | |  |  |  |  |  |  |  |
| -tumour sample |  |  |  |  |  |  | $ - |  |
| -blood sample | MBS | MBS13839 | 1.00 | $ 23.05 | $ 23.05 | 1.00 | $ 23.05 |  |
| -courier/freight to lab |  |  |  | $ 2.00 | $ 2.00 | 1.00 | $ 2.00 |  |
|  |  |  |  |  |  |  | $ 25.05 |  |
|  |  |  |  |  |  |  | Subtotal | **$ 250.50** |
| **2.DNA extraction** | |  |  |  |  |  |  |  |
| FFPE Extraction pre-processing (autmated - MilliSect) tips | AVENIO Millisect | e.g 08106507001 | 48 | pack | $ 50.00 | 48 | $ 1.04 |  |
| QiaSymphony DNA Tissue Extraction (per sample) |  |  |  |  |  |  |  |  |
| DNA Midi Kit (96) | Qiagen | 937255 | 1 | sample | $ 1,193.00 | 96 | $ 8.70 |  |
| Sample Cartridge (336) | Qiagen | 997002 | 9 | units | $ 193.00 | 336 | $ 3.10 |  |
| Rod Cover (144) | Qiagen | 997004 | 2 | units | $ 135.00 | 114 | $ 1.42 |  |
| Filter Tips 200 (1024) | Qiagen | 990332 | 2 | tips | $ 123.00 | 1024 | $ 0.14 |  |
| Filter Tips 1500 (1024) | Qiagen | 997024 | 55 | tips | $ 169.00 | 1024 | $ 5.45 |  |
| Buffer ATL (200 ml) | Qiagen | 939016 | 0.22 | ml | $ 135.00 | 200 | $ 0.13 |  |
| Proteinase K (2 ml) | Qiagen | 19131 | 0.02 | ml | $ 160.00 | 2 | $ 1.20 |  |
| QiaSymphony DNA Blood Extraction (per sample) |  |  |  |  |  |  |  |  |
| DNA Midi Kit (96) | Qiagen | 937255 | 1 | sample | $ 1,193.00 | 96 | $ 8.70 |  |
| Sample Cartridge (336) | Qiagen | 997002 | 9 | units | $ 193.00 | 336 | $ 3.10 |  |
| Rod Cover (144) | Qiagen | 997004 | 2 | units | $ 135.00 | 114 | $ 1.42 |  |
| Filter Tips 200 (1024) | Qiagen | 990332 | 2 | tips | $ 123.00 | 1024 | $ 0.14 |  |
| Filter Tips 1500 (1024) | Qiagen | 997024 | 55 | tips | $ 169.00 | 1024 | $ 5.45 |  |
| -consumables |  |  |  |  |  |  | $ 39.99 | $ 399.92 |
| Scientist - QiaSymphony | 0.7 days per 12 samples |  | 0.875 | hours | $ 46.57 | $ 40.75 | $ 40.75 |  |
| -labour |  |  |  |  |  |  | $ 81.50 | $ 815.01 |
| Genomic DNA TapeStation assay | TapeStation QC | 5067-5365 | 1 | sample | $ 6.63 | 1 | $ 4.62 |  |
| DropPlate 96-S (10 plates) | Trinean QC | 210096-S | 0.01 | plate | $ 46.20 | 1 | $ 0.51 |  |
| Scientist - QC and submission | 1 day per 12 samples |  | 0.625 | hours | $ 46.57 |  | $ 29.11 |  |
|  |  |  |  |  |  |  | $ 63.86 | $ 638.62 |
|  |  |  |  |  |  |  | Subtotal | **$ 1,853.55** |
| **3.Library preparation and Capture** | |  |  |  |  |  |  |  |
| Agencourt AMPure XP beads 450ml | Exome | A63882 | 0.2 | mL | $ 7,670.00 | 450 | $ 3.24 |  |
| Dynabeads M-270 Streptavidin (2mL) | Exome | 653-05 | 120 | ul | $ 598.00 | 2000 | $ 35.88 |  |
| xGen® Lockdown® Reagents 96 rxn | Exome | 1072281 | 1 | sample | $ 288.00 | 96 | $ 3.00 |  |
| xGen® Universal Blockers - TS Mix, 96 rxn | Exome | 1075475 | 1 | sample | $ 5,940.00 | 96 | $ 61.88 |  |
| Illumina P5 Primer | Exome | IDT p5 primer | 1 | sample | $ 6.80 | 192 | $ 0.04 |  |
| Illumina P7 Primer | Exome | IDT p7 primer | 1 | sample | $ 7.14 | 192 | $ 0.04 |  |
| xGen® Pan-Cancer Panel v1.5 96 rxn | Exome | 1056204 | 1 | sample | $ 16,560.00 | 96 | $ 124.20 |  |
| xGen® Exome Research Panel v1.0 96 rxn | Exome | 1056115 | 1 | sample | $ 30,000.00 | 96 | $ 218.75 |  |
| Human Cot-1 DNA® (500ug) | Exome | 15279-011 | 5.1 | ug | $ 281.49 | 500 | $ 2.87 |  |
| KAPA HiFi HotStart ReadyMix (500 rxn) | Exome | KP-KK2602 | 30 | ul | $ 485.00 | 6250 | $ 2.33 |  |
| Eppendorf "DNA LoBind Tube, 1.5 ml" (PK/250 CLEAN) | Exome | EPPE0030108.051 | 3 | tube | $ 27.43 | 250 | $ 0.33 |  |
| TUBES,0.2ML,LOBIND,4332 | Exome | EPPE0030124.332 | 2 | tube | $ 120.70 | 1000 | $ 0.24 |  |
| Hard Shell Skirted 96 Well PCR Plate (blue) | Exome | 6008870 | 0.15 | plate | $ 264.00 | 50 | $ 0.81 |  |
| StorPlate- 96 well V-bottom | Exome | 6008290 | 0.08 | plate | $ 152.00 | 50 | $ 0.23 |  |
| 175 uL filter non-conductive tips | Plasticware | 6000685 | 0.08 | box | $ 128.00 | 10 | $ 0.98 |  |
| 25 uL filter non-conductive tips | Exome | 6000689 | 0.08 | box | $ 136.00 | 10 | $ 1.05 |  |
| 150mL Tub (4 tips) | LCGX QC | 6000583 | 0.08 | trough | $ 28.00 | 25 | $ 0.09 |  |
| -consumables |  |  |  |  |  |  | $ 911.91 | $ 9,119.10 |
|  |  |  |  |  |  |  |  |  |
| Covaris 96 microTUBES plate with AFA fibers | KAPA Hyper Library Prep Reagent (IDT) | 520078 | 1 | tube | $ 830.00 | 96 | $ 7.35 |  |
| Agencourt AMPure XP beads 450ml | KAPA Hyper Library Prep Reagent (IDT) | A63882 | 0.3 | mL | $ 7,670.00 | 450 | $ 4.87 |  |
| KAPA Hyper Prep Kit (96 rxn) | KAPA Hyper Library Prep Reagent (IDT) | KP-KK8504 | 1 | sample | $ 4,332.87 | 96 | $ 45.13 |  |
| IDT 8x12 TS HT Dual Indices | KAPA Hyper Library Prep Reagent (IDT) | 00519392Q | 1 | sample | $ 5,966.40 | 1600 | $ 3.73 |  |
| Hard Shell Skirted 96 Well PCR Plate (blue) | Kapa Hyper Library Prep Batch Consumables & QC (Automated - per sample) | 6008870 | 0.46 | plate | $ 264.00 | 50 | $ 2.44 |  |
| 175 uL filter non-conductive tips | Kapa Hyper Library Prep Batch Consumables & QC (Automated - per sample) | 6000685 | 0.21 | box | $ 128.00 | 10 | $ 2.67 |  |
| 25 uL filter non-conductive tips | Kapa Hyper Library Prep Batch Consumables & QC (Automated - per sample) | 6000689 | 0.13 | box | $ 136.00 | 10 | $ 1.70 |  |
| MDT P235 Disposable Tips | Kapa Hyper Library Prep Batch Consumables & QC (Automated - per sample) | 6001289 | 0.03 | box | $ 768.00 | 50 | $ 0.48 |  |
| Reagent Troughs 8 tip 60ml qty50 | Kapa Hyper Library Prep Batch Consumables & QC (Automated - per sample) | 6008104 | 0.23 | trough | $ 170.00 | 50 | $ 0.78 |  |
| -consumables |  |  |  |  |  |  | $ 138.30 | $ 1,383.04 |
| QUBIT DSDNA HS ASSAY KIT (500) | Qubit QC | Q32854 | 1 | sample | $ 1.15 |  | $ 1.15 |  |
| D1000 TapeStation Assay | TapeStation QC | 5067-5582 | 1 | sample | $ 4.62 |  | $ 4.62 |  |
| -QC |  |  |  |  |  |  | $ 11.54 | $ 115.40 |
| Scientist - library prep + capture (hours) | 2.2 days per 12 samples |  | 1.375 | hours | $ 46.57 | $ 64.04 | $ 64.04 |  |
| Scientist - pre seq QC (bioA+qPCR) (hours) | 0.1 days per 6 samples |  | 0.125 | hours | $ 46.57 | $ 5.82 | $ 5.82 |  |
| -labour |  |  |  |  |  |  | $ 139.72 | $ 1,397.16 |
|  |  |  |  |  |  |  | Subtotal | **$ 12,014.70** |
| **4.Sequencing** | |  |  |  |  |  |  |  |
| NextSeq® 500/550 High Output Kit v2 (150 cycles) [2017] | NextSeq | FC-404-2002 | 6 | samples | $ 3,335.25 |  | $ 555.88 |  |
| -sequencing |  |  |  |  |  |  | $ 1,111.75 | $ 11,117.50 |
| Scientist – sequencing (hours) | 0.3 day/run (6 samples) | set up | 0.375 | hours | $ 46.57 |  | $ 17.46 |  |
|  | 0.3 day/run (6 samples) | data release | 0.375 | hours | $ 46.57 |  | $ 17.46 |  |
|  |  |  |  |  |  |  | $ 69.86 | $ 698.58 |
| -labour |  |  |  |  |  |  | Subtotal | **$ 11,816.08** |
| **5. Analysis** | |  |  |  |  |  |  |  |
| Total time of 20 minutes per run (6 samples/3 patients per run) | HEW6 |  | 0.06 | $ 51.86 | $ 2.88 | 2.88 | $ 28.81 |  |
| -informatics labour |  |  |  |  |  |  | $ 57.63 | $ 576.27 |
| Novalign (Laboratory license) | $1,280.00 | per year |  | $ 0.37 |  |  | $ 0.37 |  |
|  |  |  |  |  |  |  | $ 0.73 | $ 7.31 |
|  |  |  |  |  |  |  | Subtotal | **$ 583.58** |
| **6. Storage** |  |  |  |  |  |  |  |  |
| - cloud storage, near line per GB AU$0.0136 per month | double for tumour/blood | 400 |  | $ 391.68 |  |  | $ 39.17 | **$ 391.68** |
| - 5 years |  | 28800 |  |  |  |  |  |  |
| **7. Reporting to clinicians** | |  |  |  |  |  |  |  |
| -multidisciplinary care team meeting prep | 1 hour per patient |  |  | $ 96.54 |  |  | $ 96.54 | **$ 965.40** |
|  |  |  |  |  |  |  |  |  |
|  |  |  |  |  |  | **TOTAL** | | **$ 27,875.49** |
|  |  |  |  |  |  | **TOTAL per person** | | **$ 2,787.55** |
|  |  |  |  |  |  |  |  |  |
| **8. Capital - sequencing machine** |  |  |  |  |  |  |  |  |
| NextSeq 500 | Illumina | Acquisition cost | $360,000.00 | Throughput | 600 |  |  | $ 131.01 |
| Maintenance costs | 50% of capital cost per patient | |  |  |  |  |  | $ 65.51 |
|  |  |  |  |  |  |  |  | **$ 196.52** |
|  |  |  |  |  |  |  |  |  |
|  |  |  |  |  |  | **TOTAL with capital** | | **$ 2,918.56** |

1. Oesophageal Cancer (blood/tumour pair) (n=100)

| **Description** | **Company** | **Catalog Number** | **Units** | **Unit Price** | **Total price** | **Samples per unit** | **Price/sample** | **Price per project** |
| --- | --- | --- | --- | --- | --- | --- | --- | --- |
|  |  |  |  |  |  |  |  |  |
| **1.Sampling** | |  |  |  |  |  |  |  |
| -tumour sample |  |  |  |  |  |  | $ - |  |
| -blood sample | MBS | MBS13839 | 1.00 | $ 23.05 | $ 23.05 | 1.00 | $ 23.05 |  |
| -courier/freight to lab |  |  |  | $ 2.00 | $ 2.00 | 1.00 | $ 2.00 |  |
|  |  |  |  |  |  |  | $ 25.05 |  |
|  |  |  |  |  |  |  | Subtotal | **$ 2,505.00** |
| **2.DNA extraction** | |  |  |  |  |  |  |  |
| QIAamp DNA Mini Kit (50) **(Blood)** | Qiagen | 51104 | 1.00 | $ 338.00 | $ 338.00 | 50 | $ 6.76 |  |
| Tips 10uL | Eppendorf | 30077504 | 1.00 | $ 192.00 | $ 192.00 | 96 | $ 4.00 |  |
| Tips 20uL | Eppendorf | 30077539 | 1.00 | $ 183.60 | $ 183.60 | 96 | $ 1.91 |  |
| Tips 200uL | Eppendorf | 30077555 | 1.00 | $ 190.80 | $ 190.80 | 96 | $ 7.95 |  |
| Tips 1000uL | Eppendorf | 30077857 | 1.00 | $ 205.00 | $ 205.00 | 96 | $ 12.81 |  |
| 96-well Tube Storage Racks |  |  | 1.00 | $ 6.90 | $ 6.90 | 96 | $ 0.07 |  |
| Safe-Lock Tube 1.5 ml, Natural | Eppendorf | 30120086 | 1.00 | $ 85.98 | $ 85.98 | 100 | $ 0.86 |  |
|  |  |  |  |  |  |  | $ 34.37 | $ 3,436.67 |
| AllPrep DNA/RNA Mini Kit (50) **(Tumour)** | Qiagen | 80204 | 1.00 | $ 527.00 | $ 527.00 | 50 | $ 10.54 |  |
| Nuclease-Free Water (10 x 50 ml) | Qiagen | 129114 | 1.00 | $ 119.00 | $ 119.00 | 1000 | $ 0.12 |  |
| B-mercaptoethanol | Sigma | M6250-10ML | 1.00 | $ 48 | $ 48 | 1000 | $ 0.05 |  |
| ETHANOL ABSOLUTE 2.5L AR | Chem-Supply | EA043-2.5L | 1.00 | $ 20 | $ 20 | 2000 | $ 0.01 |  |
| Tips 10uL | Eppendorf | 30077504 | 1.00 | $ 192.00 | $ 192.00 | 96 | $ 4.00 |  |
| Tips 20uL | Eppendorf | 30077539 | 1.00 | $ 183.60 | $ 183.60 | 96 | $ 1.91 |  |
| Tips 200uL | Eppendorf | 30077555 | 1.00 | $ 190.80 | $ 190.80 | 96 | $ 7.95 |  |
| Tips 1000uL | Eppendorf | 30077857 | 1.00 | $ 205.00 | $ 205.00 | 96 | $ 12.81 |  |
| 96-well Tube Storage Racks |  |  | 1.00 | $ 6.90 | $ 6.90 | 96 | $ 0.07 |  |
| Safe-Lock Tube 1.5 ml, Natural | Eppendorf | 30120086 | 1.00 | $ 85.98 | $ 85.98 | 100 | $ 0.86 |  |
| -consumables |  |  |  |  |  |  | $ 49.82 ^1^ | $ 4,982.08 |
| Qubit 0.5 ml Tubes | ThermoFisher | Q32856 | 1.00 | $ 135.00 | $ 135.00 | 500 | $ 0.27 |  |
| Qubit dsDNA Broad Range Assay Kit | ThermoFisher | Q32853 | 1.00 | $ 168.00 | $ 168.00 | 100 | $ 1.68 |  |
| SNP arrays | Illumina |  | 1.00 | $ 68.00 | $ 68.00 | 1 | $ 88.40 |  |
| -validation |  |  |  |  |  |  | $ 180.70 | $ 18,070.00 |
| Scientist - non PhD research officer (hours) |  |  |  |  |  |  |  |  |
| - labour, 2 week to prepare assumed |  |  | 76.00 | $ 46.57 | $ 3,539.47 |  | $ 35.39 | $ 3,539.47 |
|  |  |  |  |  |  |  | Subtotal | **$ 30,028.22** |
| **3.Library preparation** | |  |  |  |  |  |  |  |
| Included in Genome.One service |  |  |  |  |  |  |  |  |
|  |  |  |  |  |  |  |  |  |
| **4.Sequencing** | |  |  |  |  |  |  |  |
| Shipping | Labcabs |  | 1.00 | $ 90.00 | $ 90.00 | 50 | $ 3.60 |  |
| Xten llumina sequencing (30 x blood 60x tumour) | The Kinghorn Cancer center/Genome.One | | Blood/tumour pair | $ 4,185.00 | $ 418,500.00 |  | $ 4,185.00 |  |
|  |  |  |  |  |  |  | $ 4,188.60 |  |
|  |  |  |  |  |  |  | Subtotal | **$ 418,860.00** |
| **5. Analysis** | |  |  |  |  |  |  |  |
| Scientist - total time of 5 weeks after return of Garvan data | WGS |  | 190.00 | $ 51.86 | $ 9,854.16 | 98.54 | $ 98.54 |  |
| -informatics labour |  |  |  |  |  |  | $ 98.54 | $ 9,854.16 |
|  |  |  |  |  |  |  | Subtotal | **$ 9,854.16** |
| **6. Storage** |  |  |  |  |  |  |  |  |
| - cloud storage, near line per GB AU$0.0136 per month | tumour=150GB | 150 | 100 | $ 14,688.00 | $ 14,688.00 |  | $ 146.88 |  |
| - 5 years | normal=72GB | 72 | 100 | $ 7,050.24 | $ 7,050.24 |  | $ 70.50 |  |
|  |  | 7350 |  |  |  |  | $ 217.38 | $ 21,738.24 |
|  |  | 529200 |  |  |  |  | Subtotal | **$ 21,738.24** |
| **7. Reporting to clinicians** | |  |  |  |  |  |  |  |
| -generating report |  |  |  |  |  |  |  |  |
| -multidisciplinary care team meeting prep | NA |  |  |  |  |  | $ - | **$ -** |
|  |  |  |  |  |  |  |  |  |
|  |  |  |  |  |  | **TOTAL** | | **$ 482,985.62** |
|  |  |  |  |  |  | **TOTAL per person** | | **$ 4,829.86** |
|  |  |  |  |  |  |  |  |  |
| **8. Capital - sequencing machine** |  |  |  |  |  |  |  |  |
| HiSeq Xten (set of 10 - priced for 1 here) | Illumina | Acquisition cost | $ 1,430,000.00 | Throughput | 5000 |  |  | $ 62.45 |
| Maintenance costs | 50% of capital cost per patient | |  |  |  |  |  | $ 31.22 |
|  |  |  |  |  |  |  |  | **$ 93.67** |
|  |  |  |  |  |  |  |  |  |
|  |  |  |  |  |  | **TOTAL with capital** |  | **$ 4,892.31** |

1. 30% extra applied for errors
2. Mesothelioma (blood/tumour pair) n=3

| **Description** | **Company** | **Catalog Number** | **Units** | **Unit Price** | **Total price** | **Samples per unit** | **Price/sample** | **Price per project** |
| --- | --- | --- | --- | --- | --- | --- | --- | --- |
|  |  |  |  |  |  |  |  |  |
| **1.Sampling** | |  |  |  |  |  |  |  |
| -tumour sample |  |  |  |  |  |  | $ - |  |
| -blood sample | MBS | MBS13839 | 1.00 | $ 23.05 | $ 23.05 | 1.00 | $ 23.05 |  |
| -courier/freight to lab |  |  |  | $ 2.00 | $ 2.00 | 1.00 | $ 2.00 |  |
| -processing / labour | n/a |  |  |  |  |  | $ 25.05 |  |
|  |  |  |  |  |  |  | Subtotal | **$ 75.15** |
| **2.DNA extraction** | |  |  |  |  |  |  |  |
| QIAamp DNA Mini Kit (50) **(Blood)** | Qiagen | 51104 | 1.00 | $ 338.00 | $ 338.00 | 50 | $ 6.76 |  |
| Tips 10uL | Eppendorf | 30077504 | 1.00 | $ 192.00 | $ 192.00 | 96 | $ 4.00 |  |
| Tips 20uL | Eppendorf | 30077539 | 1.00 | $ 183.60 | $ 183.60 | 96 | $ 1.91 |  |
| Tips 200uL | Eppendorf | 30077555 | 1.00 | $ 190.80 | $ 190.80 | 96 | $ 7.95 |  |
| Tips 1000uL | Eppendorf | 30077857 | 1.00 | $ 205.00 | $ 205.00 | 96 | $ 12.81 |  |
| 96-well Tube Storage Racks |  |  | 1.00 | $ 6.90 | $ 6.90 | 96 | $ 0.07 |  |
| Safe-Lock Tube 1.5 ml, Natural | Eppendorf | 30120086 | 1.00 | $ 85.98 | $ 85.98 | 100 | $ 0.86 |  |
|  |  |  |  |  |  |  | $ 34.37 | $ 103.10 |
| AllPrep DNA/RNA Mini Kit (50) **(Tumour)** | Qiagen | 80204 | 1.00 | $ 527.00 | $ 527.00 | 50 | $ 10.54 |  |
| Nuclease-Free Water (10 x 50 ml) | Qiagen | 129114 | 1.00 | $ 119.00 | $ 119.00 | 1000 | $ 0.12 |  |
| B-mercaptoethanol | Sigma | M6250-10ML | 1.00 | $ 48 | $ 48 | 1000 | $ 0.05 |  |
| ETHANOL ABSOLUTE 2.5L AR | Chem-Supply | EA043-2.5L | 1.00 | $ 20 | $ 20 | 2000 | $ 0.01 |  |
| Tips 10uL | Eppendorf | 30077504 | 1.00 | $ 192.00 | $ 192.00 | 96 | $ 4.00 |  |
| Tips 20uL | Eppendorf | 30077539 | 1.00 | $ 183.60 | $ 183.60 | 96 | $ 1.91 |  |
| Tips 200uL | Eppendorf | 30077555 | 1.00 | $ 190.80 | $ 190.80 | 96 | $ 7.95 |  |
| Tips 1000uL | Eppendorf | 30077857 | 1.00 | $ 205.00 | $ 205.00 | 96 | $ 12.81 |  |
| 96-well Tube Storage Racks |  |  | 1.00 | $ 6.90 | $ 6.90 | 96 | $ 0.07 |  |
| Safe-Lock Tube 1.5 ml, Natural | Eppendorf | 30120086 | 1.00 | $ 85.98 | $ 85.98 | 100 | $ 0.86 |  |
| -consumables |  |  |  |  |  |  | $ 38.32 | $ 114.97 |
| Qubit 0.5 ml Tubes | ThermoFisher | Q32856 | 1.00 | $ 135.00 | $ 135.00 | 500 | $ 0.27 |  |
| Qubit dsDNA Broad Range Assay Kit | ThermoFisher | Q32853 | 1.00 | $ 168.00 | $ 168.00 | 100 | $ 1.68 |  |
| SNP arrays | Illumina |  | 1.00 | $ 68.00 | $ 68.00 | 1 | $ 68.00 |  |
| -validation |  |  |  |  |  |  | $ 139.90 | $ 419.70 |
| Scientist - non PhD RO |  |  |  |  |  |  |  |  |
| - labour, 2 hours to prepare |  |  | 2.00 | $ 46.57 | $ 93.14 |  | $ 31.05 | $ 93.14 |
|  |  |  |  |  |  | Subtotal |  | **$ 730.92** |
| **3.Library preparation** | |  |  |  |  |  |  |  |
| Included in Garvan service |  |  |  |  |  |  |  |  |
|  |  |  |  |  |  |  |  |  |
| **4.Sequencing** | |  |  |  |  |  |  |  |
| BGI sequencing | 30 x blood 60x tumour | | Blood/tumour pair | $ 1,631.26 | $ 4,893.78 |  | $ 1,631.26 |  |
|  |  |  |  |  |  |  | $ 1,631.26 | **$ 4,893.78** |
| **5. Analysis** | |  |  |  |  |  |  |  |
| Total time of 6 days after return of BGI data | WGS |  | 45.00 | $ 51.86 | $ 2,333.88 | 2333.88 | $ 777.96 |  |
| -informatics labour |  |  |  |  |  |  | $ 777.96 | $ 2,333.88 |
|  |  |  |  |  |  |  | Subtotal | **$ 2,333.88** |
| **6. Storage** |  |  |  |  |  |  |  |  |
| - cloud storage, near line per GB AU$0.0136 per month | tumour=150GB | 150 | 3 | $ 440.64 | $ 440.64 | n/a | $ 146.88 |  |
| - 5 years | normal=72GB | 72 | 3 | $ 211.51 | $ 211.51 |  | $ 70.50 |  |
|  |  | 366 |  |  |  |  | $ 217.38 | $ 652.15 |
|  |  | 26352 |  |  |  |  | Subtotal | **$ 652.15** |
| **7. Reporting to clinicians** | |  |  |  |  |  |  |  |
| -generating report |  |  |  |  |  |  |  |  |
| -multidisciplinary care team meeting prep | NA |  |  |  |  |  | $ - | **$ -** |
|  |  |  |  |  |  | **TOTAL** | | **$ 8,685.87** |
|  |  |  |  |  |  | **TOTAL per person** | | **$ 2,895.29** |
|  |  |  |  |  |  |  |  |  |
| **8. Capital - sequencing machine** |  |  |  |  |  |  |  |  |
| BGISEQ | BGI | Acquisition cost | $ 340,000.00 | Throughput | 1000 |  |  | $ 74.24 |
| Maintenance costs | 50% of capital cost per patient | |  |  |  |  |  | $ 37.12 |
|  |  |  |  |  |  |  |  | **$ 111.36** |
|  |  |  |  |  |  |  |  |  |
|  |  |  |  |  |  | **TOTAL with capital** |  | **$ 2,969.53** |
