# Supplementary material for: Estimating the costs of genomic sequencing in cancer control
Source: BMC Health Serv Res. 2020 Jun 3;20:492. doi: 10.1186/s12913-020-05318-y (PMC7268398; doi:10.1186/s12913-020-05318-y)
Supplement: Supplementary file 2 — Additional file 2. Features of sequencing that will influence costs. [file 12913_2020_5318_MOESM2_ESM.docx]

**Appendix 2: Features of sequencing that will influence costs**

- *Purpose of sequencing:* the purpose of sequencing can influence the extent of resources required and differ if the intent is risk prediction (germline deoxyribose nucleic acid (DNA) required only) or identifying tumour pathogenic variations (requiring both germline and tumour DNA sequencing);
- *Sequencing method:* whole genome sequencing is the most comprehensive sequencing method and is costlier than whole exome sequencing, which covers only protein-coding regions of the genome or 1-2% of the full genome. In turn, whole exome sequencing is more costly than targeted panels which test for a panel of known cancer genes. These differences in cost are largely due to the specific sequencing chemistry requirements for each application on specific instruments;
- *Sequencing platforms:* the latest sequencing systems are faster, smaller and increase throughput, that is, the number of samples per run. Illumina is the market leader having at least 80% market share of sequencing platforms with several new sequencers released over the last five years (NextSeq™ 500 in 2014, HiSeq Xten™ in 2014, BGISEQ-500™ in 2015 and NovaSeq™ in 2017). However, regardless of sequencing platform, higher sample numbers will mean economies of scale are possible.
- *Reading depth/coverage*: will depend on the sequencing purpose; however while lower sequencing depth is less expensive and quicker, it might affect the quality of the sequencing outcomes;
- *Automation:* costs are reduced when robotics or other forms of automation are used, and the decreased dependence on human resources results in potentially quicker sequencing, at the expense of increased maintenance costs;
- *Extent of checking, validation and confirmatory testing*: different protocols may be followed to execute sequencing workflows and the addition of pre- and post- sequencing tests (e.g., quantitative polymerase chain reaction testing, mRNA sequencing, fluorescence in situ hybridization, immunohistochemical tests) to validate the findings adds to the cost of sequencing;
- *Supplier prices*: the costs of consumables involved in sequencing or commercial sequencing services offered depend on industry quotes, discounts offered, number of samples and exchange rates may also change prices.
- *Services outsourced*: Commercial services can include a mix of resources e.g., freight, DNA extraction, library preparation, analysis or individual sequencing components as governed by accredited protocols within the laboratories.
- *Bioinformatic analytical experience*: staffing costs vary depending on the seniority of the bioinformatics specialist involved and their relative skill and speed at interpreting genomic information for the clinical report.
- *Data storage:* requirements for data storage differ according to commercial quotes, the duration of storage, the size of data and the frequency of use expected (retrieval and re-analysis requirements).
