# Supplementary material for: Estimating the costs of genomic sequencing in cancer control
Source: BMC Health Serv Res. 2020 Jun 3;20:492. doi: 10.1186/s12913-020-05318-y (PMC7268398; doi:10.1186/s12913-020-05318-y)
Supplement: Supplementary file 3 — Additional file 3. Calculation of annual per person cost of sequencers. [file 12913_2020_5318_MOESM3_ESM.docx]

**APPENDIX 3: Calculation of annual per person cost of sequencers**

| **Project** | **Platform** | **Acquisition cost USD*** | **USD per unit** | **AUD per unit^1^** |  | **Capital outlay (rounded) AUD** | **r** | **n (years)** | **Discount factor** | **Annuity** | **E** | **Annual throughput (patients)** | **Capital Cost per patient** |
| --- | --- | --- | --- | --- | --- | --- | --- | --- | --- | --- | --- | --- | --- |
|  |  |  |  |  |  |  |  |  |  |  |  |  |  |
| Lung/Mel & breast | NextSeq 500 | $250,000 | $250,000 | $357,438 |  | $ 360,000 | 3% | 5 | 0.862609 | 4.57971 | $ 78,608 | 3500 | **$ 22.46** |
|  |  |  |  |  |  |  |  |  |  |  |  |  |  |
| Lung cancer | NextSeq 500 | $250,000 | $250,000 | $357,438 |  | $ 360,000 | 3% | 5 | 0.862609 | 4.57971 | $ 78,608 | 600 | **$ 131.01** |
|  |  |  |  |  |  |  |  |  |  |  |  |  |  |
| Oesophageal | HiSeq Xten | $10,000,000 (set of 10) | $1,000,000 | $1,429,750 |  | $ 1,430,000 | 3% | 5 | 0.862609 | 4.57971 | $ 312,247 | 5000 | **$ 62.45** |
|  |  |  |  |  |  |  |  |  |  |  |  |  |  |
| Melanoma | NovaSeq | $985,000 | $985,000 | $1,408,304 |  | $ 1,410,000 | 3% | 5 | 0.862609 | 4.57971 | $ 307,880 | 5000 | **$ 61.58** |
|  |  |  |  |  |  |  |  |  |  |  |  |  |  |
| Mesothelioma | BGISEQ-500 | $240,000 | $240,000 | $343,140 |  | $ 340,000 | 3% | 5 | 0.862609 | 4.57971 | $ 74,241 | 1000 | **$ 74.24** |
|  |  |  |  |  |  |  |  |  |  |  |  |  |  |
| *Source: | <https://docs.google.com/spreadsheets/d/1GMMfhyLK0-q8XkIo3YxlWaZA5vVMuhU1kg41g4xLkXc/edit?hl=en_GB&hl=en_GB#gid=1569422585> | | | | | | | | | | |  |  |

1. AUD 1.42975 = US 1.0

**Annual equivalent cost**

K= capital outlay

$${K=E \frac{1-(1+r)}{r}}^{-n}$$

E = annual equivalent cost

r= interest rate

n=useful life of equipment
